# Estrogen Receptor-Beta Gene Polymorphism in women with Breast Cancer at the Imam Khomeini Hospital Complex, Iran

**DOI:** 10.1186/1471-2350-11-109

**Published:** 2010-07-07

**Authors:** Sakineh Abbasi

**Affiliations:** 1 Department of Medical Laboratory Sciences, Faculty of Allied Medicine, Tehran University of Medical Sciences, Iran

## Abstract

ER-alpha and ER-beta genes have been proven to play a significant role in breast cancer. Epidemiologic studies have revealed that age-incidence patterns of breast cancer in Middle East differ from those in the Western countries. Two selected coding regions in the ER-β gene (exons 3 and 7) were scanned in Iranian women with breast cancer (150) and in healthy individuals (147). PCR single-strand conformation polymorphism was performed. A site of silent single nucleotide polymorphism was found only on exon 7. The SNP was found only in breast cancer patients (5.7%) (χ^2 ^= 17.122, *P *= 0.01). Codon 392 (C1176G) of allele 1 was found to have direct association with the occurrence of lymph node metastasis. Our data suggest that ER-β polymorphism in exon 7 codon 392 (C1176G) is correlated with various aspects of breast cancer and lymph node metastasis in our group of patients.

## Introduction

Breast cancer is the most prevalent cancer among women in most parts of the world, including Iran. Geographical variations in incidence and mortality rates of breast cancer suggest that the known risk factors for breast cancer may vary in different parts of the world and that environmental factors may be of greater importance than genetic factors [1]. For instance, in Iran it has been shown that, even after adjusting for age, young women are at relatively higher risk for developing breast cancer than are their Western counterparts [2,3].

It is known that breast cancer typically arises in luminal epithelial cells of the mammary gland [4,5]. These cells contain estrogen receptors (ERs), which respond to ovarian estrogen in normal mammary gland development. How estrogens stimulate cell growth is not fully understood, but it is known that estrogen activation of ERs results in transcription of various genes that are involved in cellular proliferation

Only a small fraction (< 5%) of women diagnosed with breast cancer have a clear hereditary predisposition [6-8], and of these, about one half have predisposing mutations in *BRCA1*, *BRCA2*, *PTEN*, *TP53*, or other known cancer predisposing genes. However, twin studies indicate that the heritability of breast cancer is about 30% [9], suggesting that genes other than the well-mapped regions act as modifiers of breast cancer risk. Although it is likely that low penetrance as well as high penetrance genes may be involved in the etiology, it remains unclear which genomic regions and which biochemical functions or signal transduction pathways account for the additional heritability of breast cancer incidence or progression.

Epidemiologic evidence suggests that estrogen plays a crucial role in most breast cancers in women who have both early menarche and late menopause. Obesity is also associated with breast cancer risk; estrogen synthesis in adipose tissue is proposed to account for this increase in risk. There is data on the involvement of estrogen receptor in the molecular processes implicated in the rise of breast cancer incidence [10-16].

ER-α polymorphic variants have been associated with breast cancer risk [17-25] in Caucasians. Furthermore, ER-α has been studied extensively, but supporting evidence is required for proving the involvement of ER-β in breast cancer [26-33]. Currently, the literature contains sparse information regarding ER-β gene expression, mutation frequency, and allelic variation in breast cancer. Thus, the present study aimed to identify candidate steroid hormone receptor gene variants in randomly selected regions (exon 3 and exon 7) of the ER-β gene that might be associated with risk of breast cancer. It is our long-term objective to establish a genetic polymorphism database for the ER-β encoding regions of the Iranian (Asian-Caucasian) genome, and to test for any correlation between ER-β polymorphism and various clinically observable features of breast cancer in Iranian women.

## Methods

### Study population

A case-control study was conducted from April 2004 to January 2008 in Tehran, Iran. The breast cancer patients (***n ***= 150; mean age 47.49 ± 11.43 years) were newly diagnosed and mostly living in Tehran. They were entered into the study if they had a confirmed pathological breast cancer diagnosis at the Imam Khomeini Hospital Complex (a large teaching and general hospital in the central district of Tehran) and were referred to the several clinics of the Cancer Institute of the hospital. The control group (***n ***= 147; median age 40.75 ± 10.54 years) included healthy women neither with any history of breast cancer nor any other neoplastic diseases, and none of their relatives had a history of breast cancer diagnosed at the same clinics [34]. Women with hysterectomy and artificial menopause, or women exposed to any kind of radiation and chemotherapy in their lifetime were excluded from the study. All participants in our study provided written informed consent to participate before entering the present study.

Demographical and risk factor data were collected using a short structured questionnaire, including information on age, weight, height, race, religion, marital status, number of pregnancies and children, age at first child birth, average lactation term, family history of breast cancer (first-degree relatives), age at menarche, age at marriage, menopausal status, and age at menopause, ABO and Rhesus blood groups, race, age at onset, lymph node metastases, cancer stage at the time of testing and ER expression in breast cancer tissue. A protocol to collect and store blood samples for future genomic tests had been approved by the institutional review board. Peripheral blood was collected and genotyping analysis was performed.

### Screening for ER-β variants by single strand conformation polymorphism analysis

In order to identify any change in the two selected exons (exons 3 and exon 7) of ER-β, PCR and SSCP was performed. Genomic DNA was extracted from blood leukocytes using the DNG™-Plus extraction kit (Cinnagen Inc, Tehran, Iran) in accordance with the manufacturer's instructions. Specific set of primers for ER-β gene exons 3 and 7 were designed by *primer3 *(v. 0.4.0) software (Table 1).

**Table 1:** Primers selected for Exons #3 and #7 of Gene ER-β

Polymorphism site	Melting temperature (°C)	Oligonucleotic sequences	Sequence size
Exon 3	59.23 59.86	Primers for PCR reaction **F **TTGCTCCCTAGAGAGACACTGA **R **CTTCACACGACCAGACTCCA	151
Exon 7	60.04 60.14	Primers for PCR reaction **F **5' GATGAGGGGAAATGCGTAGA 3' **R **5' GGCCCAGCTGTGTGATTACT 3'	156

PCR was performed through 30 cycles with the following steps: denaturation 30 sec at 95°C, annealing 30 sec at 58°C and elongation 40 seconds at 72°C. The SSCP was carried out on 12% polyacrylamide gel (29:1 Acrylamide/Bisacrylamide), with 200 V, for 20 h at 16°C. After electrophoresis, silver nitrate staining was used for visualizing the bands. To identify and confirm the exhibited irregular pattern in SSCP, PCR samples demonstrating varying band shift patterns as the result of first sequencing with forward primer, were re-purified on agarose gel using a DNA Extraction Kit (Fermentas # K0153, Germany) and directly sequenced by big dye Terminator V3.1 Cycle Sequencing kit protocol, (Applied Biosystem Kit, Microgen Co., USA), on a sequencer ABI 3130XL (16-capillaries).

The PCR product purification method was used in order to confirm sequencing by reverse primer. The PCR products were purified using QIAquick PCR purification Kit (50) (QIAGEN, USA).

### Statistical analysis

χ^2 ^testing was employed to assess the influence of polymorphism status on features of breast cancer. Unconditional logistic regression analysis was performed using SPSS software (version 11.5 for Windows XP; SPSS Inc., Cary, NC, USA) to calculate odds ratios (ORs) with 95% confidence intervals (CIs) and to examine the predictive effect of each factor on risk for breast cancer. *P *< 0.05 was considered as statistically significant.

## Results

In this study, 150 breast cancer patients and 147 healthy women without family history of breast cancer were selected as controls. In exon #3, we did not find novel mutation or any synonymous SNP in the Iranian population. Exon #7 revealed no novel mutations but a silent SNP in codon 392 (Leu 392 Leu), **rs1256054 **(dbSNP128), a conversion of nucleotide C to G (C 1176 G) was observed. Both CTC and CTG code for Leucine amino acid. The observed numbers of individuals with different genotypes showed that this SNP fitted the Hardy-Weinberg equilibrium for both control and patient groups (***P ***> 0.05). The codon 392 polymorphism was found only in patients. Statistically significant frequency was achieved; 8.7% for the CG genotype, and 1.3% for the GG genotype in comparison to 90.0% CC in the normal genotype (χ^2 ^= 4.769, *P *= 0.029) (Table 2).

**Table 2:** Genotypic distribution frequencies of codon 392 in exon 7 of estrogen receptor-β gene in the study population: breast caner versus control groups

Codon 392	Normal^a^		Heterozygote^b^		Homozygote^c^		Total		Test result
	Frequency	Percent	Frequency	Percent	Frequency	Percent	Frequency	Percent	
Case	135	90.0	13	8.7	2	1.3	150	100	χ^2 ^= 4.769 *P *= 0.029
Control	147	100	-	-	-	-	147	100	
**Total**	282	94.9	13	4.4	2	0.7	297	100	

^a ^Genotype normal or 329CC, CTC/CTC, ^b ^Genotype heterozygote or 329CG, CTC/CTG, ^c ^Genotype homozygote or 329GG, CTG/CTG

Among all risk factors, only race and ABO blood groups were statistically significant (*P*< 0.05) between cases and controls. Women from the Fars province (near the southern part of Iran) revealed a significant genotypic distribution frequency only within CG heterozygote patients (χ^2 ^= 9.11, *P *= 0.003). When the ABO blood groups were considered, A and B blood groups showed statistically significant frequencies between cases and controls (χ^2 ^= 4.920, *P *= 0.027, and χ^2 ^= 5.108, *P *= 0.024 respectively) (Table 3).

**Table 3:** Genotypic distribution frequencies of codon 392 in exon 7 of estrogen receptor-β gene and major risk factors in study population: breast cancers, versus control groups

Characteristic	Group	00^a ^%	01^b ^%	11^c ^%	Test result
Age at menarche(years)					
</= 12	Case	83.3	15.0	1.7	χ^2 ^= 1.887 *P *= 0.169
	control	100	-	-	
	total	89.6	9.4	1.0	
> 12	Case	94.4	4.4	1.1	χ^2 ^= 2.229 *P *= 0.135
	control	100	-	-	
	total	97.5	2.0	0.5	
**Race**					
Arab & Armani	Case	100	-	-	-
	control	-	-	-	
	total	100	-	-	
Fars	Case	90.0	10.0	-	χ^2 ^= 9.11 *P *= 0.003
	control	100	-	-	
	total	95.9	4.1	-	
Lor & Kurdish	Case	83.3	11.1	5.6	χ^2 ^= 0.697 *P *= 0.404
	control	100	-	-	
	total	88.9	7.4	3.7	
Turkish	Case	93.5	4.3	2.2	χ^2 ^= 1.17 *P *= 0.279
	control	100	-	-	
	total	96.5	2.4	1.1	
Gilaki & Mazani	Case	87.0	13.0	-	χ^2 ^= 1.527 *P *= 0.217
	control	100	-	-	
	total	91.2	8.8	-	
**ABO blood Groups**					
A	Case	88.9	11.1	-	χ^2 ^= 4.920 *P *= 0.027
	control	100	-	-	
	total	95.7	4.3	-	
B	Case	85.7	14.3	-	χ^2 ^= 5.106 *P *= 0.024
	control	100	-	-	
	total	95.9	4.1	-	
AB	Case	100	-	-	-
	control	100	-	-	
	total	100	-	-	
O	Case	90.3	7.8	1.9	χ^2 ^= 1.884 *P *= 0.17
	control	100	-	-	
	total	93.6	5.1	1.3	

^a ^Genotype normal or 329CC, CTC/CTC, ^b ^Genotype heterozygote or 329CG, CTC/CTG, Genotype homozygote or 329GG, CTG/CTG

Among breast cancer patients, 70.1% revealed a first-degree family history of breast cancer with CG (52.6%) or GG (10.5%) genotype and 2.3% without family history of breast cancer with CG genotype. This difference was statistically significant (χ^2 ^= 27.645, *P *= 0.001). In breast cancer patients with LN metastasis, the genotypic distribution frequency of codon 392 showed statistically significant difference with frequencies of 30.4% for CG and 8.7% for GG, in comparison with breast cancer patients without LN metastases (4.7%) (χ^2 ^= 17.314, *P *= 0.001) (Table 4).

**Table 4:** Genotypic distribution frequencies of codon 392 in exon 7 of estrogen receptor-β gene and selected demographic characteristics and major risk factors

Characteristic	00^2 ^%	01^b^%	11^c ^%	Test result
**BMI (kg/m^2^)**				
</= 18.5	80.0	20.0	-	χ^2 ^= 0.144 *P *= 0.705
18.5-24.9	91.2	7.0	1.8	
25-29.9	90.9	9.1	-	
> 30	87.9	9.1	3.0	
**Total**	90.0	8.7	1.3	
**Onset age of breast cancer (years)**				
</= 40	91.7	8.3	-	χ^2 ^= 0.959 *P *= 0.328
> 40	89.2	8.8	2.0	
**Total**	90.0	8.7	1.3	
**Family history of breast cancer**				
First-degree family affected	36.8	52.6	10.5	χ^2 ^= 27.645 *P *= 0.001
Not affected	97.7	2.3	-	
**Total**	90	8.7	1.3	
**Lymph node metastases**				
Yes	60.9	30.4	8.7	χ^2 ^= 17.314 *P *= 0.001
No	95.3	4.7	-	
**Total**	90.0	8.7	1.3	
**ER expression in breast cancer tissue**				
Positive	80.0	15.0	5.0	χ^2 ^= 4.799 *P *= 0.028
Negative	94.6	5.4	-	
Not studied	88.9	11.1	-	
**Total**	90.0	8.7	1.3	

^a ^Genotype normal or 329CC, CTC/CTC, ^b ^Genotype heterozygote or 329CG, CTC/CTG, **^c ^**Genotype homozygote or 329GG, CTG/CTG

The frequency of allele 392G was significantly (*χ^2^*= 4.583, *P *= 0.032) higher in cancer patients with the age at menarche being 12 years old or below (9.2%) than those reaching menarche above 12 years of age (3.3%). The frequency of allele 392G was significantly higher in cancer patients with a first-degree family history (36.8%) than in those without a family history (1.1%, with a difference of 35.7%), in comparison with the frequency of allele 392C which is significantly lower in cancer patients with a first-degree family history than in those without a family history (*χ^2 ^*= 78.847, *P *= 0.001) (Table 5). Moreover, the allelic frequency of 392C was significantly higher in cancer patients with LN metastases (23.9%) than in those without LN metastases (2.4%) with a difference of 21.5%, in comparison with the frequency of allele 392 C which was significantly lower in cancer patients with LN metastases (76.1%) than in those without LN metastases (97.6%) (*χ^2 ^*= 33.838, *P *= 0.001) (Table 5).

**Table 5:** Allelic frequencies of estrogen receptor-β exon 7, codon 392 (CTC/CTG) in the study population: breast cancer cases versus control groups and breast cancer cases in the presence versus the absence of major risk factors

		ER-β Alleles	
Characteristic		C^a^	G^b^
**Breast cancer**			
Case	(n = 150)	283(94.3%)	17(5.7%)
Control	(n = 147)	294(100%)	-
		χ^2 ^= 17.122, *P *= 0.001	
**Age at menarche(years)**			
</= 12	(n = 60)	109(90.8%)	11(9.2%)
> 12	(n = 90)	174(96.7%)	6(3.3%)
		χ^2 ^= 4.583, *P *= 0.032	
**Age at menopause (years)**			
=< 50	(n = 47)	87(92.6%)	7(7.4%)
> 50	(n = 12)	24(100%)	-
		χ^2 ^= 1.884, *P *= 0.17	
**ABO blood groups**			
A	(n = 27)	51(94.4%)	3(5.6%)
B	(n = 14)	26(92.9%)	2(7.1%)
AB	(n = 6)	12(100%)	-
O	(n = 103)	194(94.2%)	12(5.8%)
		χ^2 ^= 0.058, *P *= 0.81	
**Race**			
Arab & Armani	(n = 3)	6(100%)	-
Fars	(n = 60)	114(95.0%)	6(5.0%)
Lor & Kurdish	(n = 18)	32(88.9%)	4(11.1%)
Turkish	(n = 46)	88(95.7%)	4(4.3%)
Gilaki & Mazani	(n = 23)	43(93.5%)	3(6.5%)
		χ^2 ^= 0.05, *P *= 0.822	
**Family history of breast cancer**			
First-degree family affected	(n = 19)	24(63.2%)	14(36.8%)
Not affected	(n = 131)	259(98.9%)	3(1.1%)
		χ^2 ^= 78.847, *P *= 0.001	
**Lymph node metastases**			
Yes	(n = 23)	35(76.1%)	11(23.9%)
No	(n = 127)	248(97.6%)	6(2.4%)
		χ^2 ^= 33.838, *P *= 0.001	
**ER expression in breast cancer tissue**			
Positive	(n = 40)	70(87.5%)	10(12.5%)
Negative	(n = 92)	179(97.3%)	5(2.7%)
Not studied	(n = 18)	34(94.4%)	2(5.6%)
		χ^2 ^= 5.161, *P *= 0.023	

^a ^Allele 392C, CTC, ^b ^Allele 392G, CTG

Significant correlation was found for first-degree relatives with family history of breast cancer and LN metastasis, as indicated by the ORs presented in Table 6. In regards to the criterion of breast cancer, genotype frequencies exhibited a significantly different distribution in cases and controls (***P ***= 0.029). The estimated risk was higher in cases (47.9%) than in controls (52.1%) in individuals with the normal CC, genotype. Nevertheless, CG or GG genotype in codon 392 was observed only in breast cancer patients.

**Table 6:** Estimated risk for selected demographic characteristic and major risk factors with estrogen receptor-β exon 7, codon 392 in different genotypes

Breast cancer	Yes	No	*P *value	OR (95% CI)
Genotype	n = 150	n = 147		
Normal^a^	135(47.9%)	147(52.1%)	0.029	1.0(reference)
Heterozygote^b^	13(100%)	-		-
Homozygote^c^	2(100%)	-		-
				
**Age at menarche (years)**	**</= 12**	**> 12**	***P *value**	**OR (95% CI)**
**Genotype**	**n = 60**	**n = 90**		
Normal	50(37.0%)	85(63%)	0.437	1.0(reference)
Heterozygote	9(69.2%)	4(30.8%)		0.261(0.077-0.893)
Homozygote	1(50%)	1(50%)		0.588(0.036 -9.613)
				
**First- degree family history of breast cancer**	**Affected**	**Not affected**	***P *value**	**OR (95% CI)**
**Genotype**	**n = 19**	**n = 131**		
Normal	7(5.2%)	128(94.8%)	0.001	1.0(reference)
Heterozygote	10(76.9%)	3(23.1%)		0.016(0.004- 0.073)
Homozygote	2(100%)	-		-
				
**Lymph node metastases**	**Yes**	**No**	***P *value**	**OR (95% CI)**
**Genotype**	**n = 23**	**n = 127**		
Normal	14(10.4%)	121(89.6%)	0.001	1.0(reference)
Heterozygote	7(53.8%)	6(46.2%)		0.099(0.029 - 0.337)
Homozygote	2(100%)	-		-

^a ^Genotype normal or 329CC, CTC/CTC, ^b ^Genotype heterozygote or 329CG, CTC/CTG, ^c ^Genotype homozygote or 329GG, CTG/CTG

Furthermore, genotype frequencies exhibited different distributions in the presence or absence of a first-degree relative family history of breast cancer, with statistical significance for codon 392 (***P ***= 0.001) and LN metastasis. The estimated risk was very much lower for both CG individuals with family history of breast cancer LN metastasis than controls (OR 0.016 and 0099 respectively, 95% CI). Moreover, GG genotype was only observed in individuals with family history of breast cancer and LN metastasis (Table 6).

## Discussion

ER-β is one of the most important players in the mechanism of estrogen action and is found in many tissues, including the central nervous system, cardiovascular system, immune system, urogenital tract, gastrointestinal tract, kidneys and lungs. It is an obvious candidate gene to harbor allelic variants which predispose to breast cancer [35]. There are several studies examining the association of ER-β variants and breast cancer risk in different populations [26-33], but these studies do not include **rs1256054**, with the exception to Zheng *et al*. [29]. This is the first study focusing on genetic variations of the ER-β gene in breast cancer patients in Iran. Two single nucleotide polymorphisms in the ER-α gene in relation to breast cancer were studied before [24,40]. In the present study, the SNPs are detected only in the cancer patient group, with significant (*P *= 0.029) increase of CG genotype (8.7%) as compared with the GG genotype (1.3%), suggesting the CG genotype confers risk for development of breast cancer (Table 2). Furthermore, the frequency of the CG genotype was found to be significantly (*P *< 0.005) increased in the Fars race and also in both blood groups A and B in comparison with the other risk factors (10%, 11.1% and 14.3% respectively) indicating that both CG genotype and mentioned characteristics might have influence in developing breast cancer (Table 3). SNPs in different regions of the ER-β gene previously reported suggest the associate of ER-β polymorphisms with breast cancer, anorexia nervosa, Parkinson and ovulatory dysfunction [26-28,36-39].

Increased frequency of CG genotype was also observed in patients with first-degree relative family history of breast cancer as compared to patients with no family history of breast cancer (a difference of 50.3%) in compare with GG genotype (a difference of 10.5%) suggesting that CG genotype might influence development of familial breast cancer more than the GG genotype. When LN status of the patients were considered again, the frequency of CG genotype was 21.7% greater in patients with LN metastasis than those without LN metastasis in comparison with the frequency of GG genotype (a difference of 8.7%), indicating that CG genotype might associate with LN metastasis in Iranian population (Table 4).

Some studies such as investigations conducted in Shanghai [29], Sweden [30] and the US [31] found no overall statistically significant differences between the allelic frequencies of cases and controls. However, a number of investigations conducted in different populations such as Finland [32], India [26], Germany [33] and Japan [37] have found a statistically significant difference between the allelic frequencies of cases and controls as we found here in our study. The results strengthened the association between codon 392 polymorphism and breast cancer development. Furthermore, we found allelic frequency of G to be significantly much higher in cases with the history of breast cancer in first-degree relatives when compared with non-familial cases (with difference of 35.7%). An increased frequency of allele G was also observed in patient with LN metastasis as compared with patients without LN metastasis (with differences of 21.5%)(*P *= 0.001) which suggest that the presence of allele G might be predictive of both familial breast cancer and bad progression of the disease (Table 5). In terms of practical utility, the relation between codon 392 variant and probability of LN metastasis deserves further consideration as a clinical indicator during pre-surgical evaluation. Such a test is of interest because lymphatic invasion is associated with local recurrence and disease progression, and LN metastasis is considered an important indicator when deciding whether chemotherapy should be given [41-44].

Nevertheless, the link between silent polymorphisms and phenotypes is unclear. One of the possibilities might be that the silent polymorphism is in linkage with another genetic mutation that directly affects breast cancer phenotype. The other possibility might be that the nucleotide composition at the silent polymorphic site could alter the gene expression level of ER-β, thus leading to LN metastasis in breast cancer.

ER-β genotypes were compared with selected clinical breast cancer features, including age at menarche, family history of breast cancer, and LN metastasis. Significant correlation (*P *= 0.001) was found for family history of breast cancer and LN metastasis, as indicated by the ORs presented in Table 6. The estimated risk was much lower for individuals with family history of breast cancer and patients with LN metastasis who were heterozygote in codon 392 (OR 0.016 and OR 0.099 respectively with 95% CI) than for the corresponding controls. This indicates that codon 392 polymorphism does not influence breast cancer risk

Taken together, codon 392 SNP might influence development of familial breast cancer and LN metastasis but it cannot be considered as a breast cancer risk factor.

## Conclusion

We have identified codon 392 CTC/CTG polymorphism to be associated with an increased risk of familial breast cancer with LN metastasis. Moreover, CG heterozygosity increases in familial breast cancer and LN metastasis. However, our results imply that although the SNP examined in this study does not play a causal role in the etiology of breast cancer, the ER-β gene variants may be involved in breast cancer susceptibility. This was the first systematic association study in ER-β variants with various aspects of breast cancer in Iran. ER-β genotype, as determined during pre-surgical evaluation, might present a surrogate marker for predicting breast cancer lymph node metastasis. Further analysis is needed to elucidate the role of ER-β polymorphisms in breast cancer susceptibility. Continuation of this study on other regions of the ER-β gene will provide additional insight into the biology and complex etiology of breast carcinogenesis.

## Abbreviations

BMI: Body Mass Index; CI: confidence interval; ESR: estrogen receptor; LN: lymph node; OR: odd ratio; PCR: polymerase chain reaction; Rh: Rhesus blood group system; SNP: single nucleotide polymorphism; SSCP: single-strand conformational

## Competing interests

The author declares that they have no competing interests.

## Pre-publication history

The pre-publication history for this paper can be accessed here:

http://www.biomedcentral.com/1471-2350/11/109/prepub
